# TEMs but not DKK1 could serve as complementary biomarkers for AFP in diagnosing AFP-negative hepatocellular carcinoma

**DOI:** 10.1371/journal.pone.0183880

**Published:** 2017-09-13

**Authors:** Liping Mao, Yueguo Wang, Delin Wang, Gang Han, Shouzhong Fu, Jianxin Wang

**Affiliations:** 1 Department of Laboratory Medicine, Affiliated Nantong No. 3 Hospital of Nantong University, Nantong, Jiangsu, China; 2 Laboratory Medicine Center, Affiliated Hospital of Nantong University, Nantong, Jiangsu, China; 3 Department of Intervention, Affiliated Nantong No. 3 Hospital of Nantong University, Nantong, Jiangsu, China; 4 Department of Liver and Gall Surgery, Affiliated Nantong No. 3 Hospital of Nantong University, Nantong, Jiangsu, China; Yonsei University College of Medicine, REPUBLIC OF KOREA

## Abstract

**Background & aims:**

Hepatitis B virus (HBV)-related hepatocellular carcinoma (HCC) is prevalent worldwide. Despite its limitations, serum alpha-fetoprotein (AFP) remains the most widely-used biomarker for the diagnosis of HCC. This study aimed to assess whether measurement of peripheral plasma Dickkopf-1 (DKK1) and Tie2-expressing monocytes (TEMs) could overcome the limitations of AFP and improve the diagnostic accuracy of HCC.

**Methods:**

Plasma DKK1 level and the percentage of TEMs in peripheral CD14+CD16+ monocytes from HCC patients (*n* = 82), HBV-related liver cirrhosis (LC) patients (*n* = 29), chronic hepatitis B (CHB) infected patients (*n* = 28) and healthy volunteers (*n* = 31) were analyzed by ELISA and flow cytometry. Receiver operating characteristic (ROC) curves were used to analyze a single biomarker, or a combination of two or three biomarkers. Univariate and multivariate analyses were performed to assess the significance of each marker in prediction of HCC and AFP-negative HCC from LC patients.

**Results:**

The percentage of TEMs in peripheral CD14+CD16+ monocytes and plasma level of DKK1 in HCC group were significantly higher than those in LC, CHB and healthy control groups (all *P*-values <0.05). The percentage of TEMs alone was also significantly higher in AFP-negative HCC group than that in LC, CHB and healthy control groups (all *P*-values <0.05). Plasma DKK1 level alone could not distinguish between AFP-negative HCC and LC patients. ROC curves showed that the optimal diagnostic cutoff value was 550.93 ng/L for DKK1 and 4.95% for TEMs. There was no significant difference in AUC of DKK1, TEMs and AFP in HCC diagnosis between the four groups (all *P*>0.05). A combination of DKK1, TEMs and AFP measurements increased the AUC for HCC diagnosis as compared with either marker alone (0.833; 95%CI 0.768–0.886). The AUC for TEMs was 0.692 (95% CI 0.564–0.819) in differentiating AFP-negative HCC from LC, with a sensitivity of 80.0% and a specificity of 65.52%. Only TEMs prevailed as a significant predictor for AFP-negative HCC differentiating from LC patients in univariate and multivariate analyses (*P* = 0.016, *P* = 0.023).

**Conclusions:**

TEMs and DKK1 may prove to be potential complementary biomarkers for AFP in the diagnosis of HCC. TEMs rather than DKK1 could serve as a complementary biomarker for AFP in the differential diagnosis of AFP-negative HCC versus LC patients.

## Introduction

Hepatocellular carcinoma (HCC) is one of the most common malignant tumors and the third leading cause of cancer-related deaths worldwide[1]. Although numerous efforts have been made to discover more reliable biomarkers for the diagnosis of HCC such asα-fetoprotein (AFP)AFP-L3, DCP and GP73, serum AFP remains the most commonly used biomarker [2–3]. However, the sensitivity and specificity of serum AFP for the diagnosis of HCC were only 39–65% and 76–94% respectively [4]. To overcome the limitations of AFP, it is necessary and urgent to find novel and more reliable serum biomarkers for early detection of HCC.

In 2005, Palmaet al [5]discovered a novel subpopulation of monocytes expressing the tyrosine kinase receptor Tie2 (tyrosine kinase with immunoglobulin and epidermal growth factor homology domains 2) as a representative surface marker. Tie2-expressing monocytes (TEMs) have been found in various human tumors to form tumor blood vessels and promote tumor angiogenesis and growth by paracrine secretion of angiogenic factors such as vascular endothelial growth factor (VEGF), basic fibroblast growth factor (b-FGF), and matrix metalloproteinase-9 (MMP-9) [6–8]. In 2013, Matsubaraet al [9] found that the frequency of TEMs, as defined as CD14+CD16+TIE2+ cells in peripheral blood, was significantly higher in HCC patients than that in non-HCC patients, and that the frequency changed with the therapeutic response or recurrence.

Dickkopf-1 (DKK-1) is a secretory antagonist of the Wnt signaling pathway. Recently, Shen et al [10] reported that serum DKK1 had better sensitivity, specificity and area under the receiver operating characteristic curve (AUC) than AFP for early diagnosis of HCC, especially in HCC patients with negative AFP results and/or those in the early stage of the disease.

In this study, we analyzed the levels of peripheral plasma DKK1 and AFP and the percentage of TEMs with respect to the sensitivity, specificity and AUC of each biomarker alone and a combination of two or three biomarkers in HCC patients with hepatitis B virus (HBV) infection and patients with HBV-related liver cirrhosis (LC), patients with chronic hepatitis B infection (CHB), and healthy controls(NC). In addition, we analyzed the diagnostic performance of either DKK1 or TEMs alone, or their combination for detection of AFP-negative HCC.

## Materials and methods

### Ethics statement

The analyses of blood samples were approved by the Research Ethics Committee of Affiliated Nantong No. 3 Hospital of Nantong University (Nantong, China). All patients and healthy controls provided their written informed consent to participate in this study.

### Sample collection and storage

HCC patients with HBV infection who were admitted to Affiliated Nantong No. 3 Hospital of Nantong University between October 2014 and June 2016 were included in this study. HCC was diagnosed based on histological findings or typical imaging characteristics as defined by the Diagnosis, Management and Treatment of Hepatocellular Carcinoma (V2011) issued by the Ministry of Health of the Chinese People’s Republic of China [11]. Tumor stages were decided according to the Barcelona Clinic Liver Cancer (BCLC) staging classification[12]. 3 mL heparinized blood was collected from each participant of the four groups, including 31 healthy volunteers (NC group), 82 HCC patients with HBV infection (HCC group), 29 patients with HBV-related liver cirrhosis (LC group), and 28 patients with chronic hepatitis B infection (CHB group). Firstly, whole blood was used for analysis of TEMs. Then, the residual whole blood was centrifugated immediately at 3000 rpm for 10 min to separate plasma, and the plasma aliquots were stored at -70°C until analysis of AFP and DKK1. Patient characteristics and disease classification are shown in Table 1. Blood samples were usually collected one day before surgery or radiofrequency ablation in the patients. Blood samples from healthy volunteers were obtained from the Health Check-up Unit, Affiliated Nantong No. 3 Hospital of Nantong University. These normal controls had normal complete blood counts, liver and kidney function tests, and had no apparent chronic inflammatory diseases.

**Table 1:** Clinical characteristics of the study participants.

Clinicopathologic characteristics	HCC	LC	CHB	NC
Number of patients	82	29	28	31
Gender (male/female)	64/18	25/4	18/10	20/11
Mean age (SD)	56.78(10.58)	52.17(13.34)	38.14(12.10)	46.30(10.63)
Child-Pugh grade (A/B/C)	71/9/2	17/10/2	25/2/1	-
Tumor size, cm (≤3/>3)	26/56	-	-	-
BCLC stage (0/A/B/C/D)	9/17/34/20/2	-	-	-
AFP, ug/L (≤20/>20)	45/37	19/10	25/3	31/0
DKK1, ng/L (≤550.93/>550.93)	9/73	11/18	14/14	21/10
TEMs, % (≤4.95/>4.95)	23/59	19/10	20/8	23/8

HCC, hepatocellular carcinoma; LC, liver cirrhosis; CHB, chronic hepatitis B virus infection; NC, normal controls

### Frequency analysis of peripheral blood TEMs

**Fig 1:**
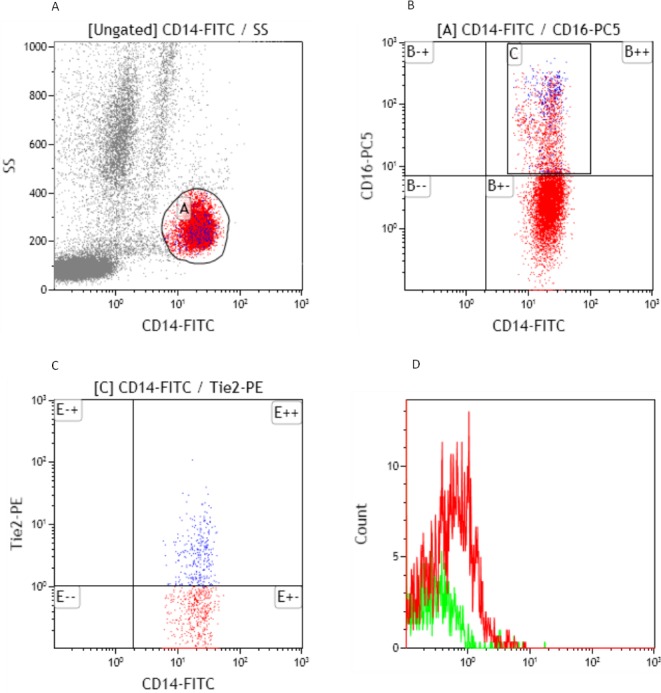
Identification of TEMs as CD14+CD16+TIE2+ cells in peripheral blood. **A.** CD14+ PBMC obtained from HCC patients were stained and analyzed by flow cytometry. **B.** CD14+ monocytes were divided into two distinct subsets, CD14+CD16+ and CD14+CD16- cells. **C.** TEMs were examined by using TIE2-PE orIgG1-k-PEisotypic antibody. **D.**The percentage of TEMs in peripheral blood CD14+CD16+ monocytes for this sample was 16.14% (the percentage of TEMs in CD14+CD16+ monocytes measured by TIE2-PE staining minus that measured by IgG1-k-PE staining, 24.2% - 8.06% = 16.14%).

Heparinized whole blood was processed at room temperature immediately after collection. TEMs were defined by their simultaneous expression of CD14, CD16 and TIE2[9]. Surface expression of CD14,CD16 and TIE2 was analyzed by direct immune-fluorescence staining followed by a lyse-wash procedure[6,13]. In brief, 100μL whole blood was blocked with 20μL human FcR blocking reagent (MACS, Miltenyi Biotec Inc. USA) for 20 min, and then incubated with fluorescence-labeled mouse anti-human Abs against CD14, CD16 and TIE2 at saturating concentrations for 20 min using CD14-FITC, CD16-PE-Cy5 (Becton-Dickinson, San Jose, CA, USA), and TIE2-PE (EMD Millipore Corporation, USA). To eliminate erythrocytes, the lyse solution was added and then wash with PBS twice. Flow cytometry was immediately performed with a Coulter Epics XL Flow Cytometer (Beckman Coulter) and Kaluza v1.2 software. IgG1-k-PE isotypic antibody (EMD Millipore Corporation, USA) was applied with all the samples as controls (Fig 1).

### Analysis of plasma DKK1 and AFP

Plasma AFP was measured using I2000 automatic chemiluminescence immunoassay analyzer (Abbott Architect i2000_SR_, USA). DKK-1 was detected using commercially available enzyme-linked immunosorbent assay (ELISA) kits according to the manufacturer’s instructions (R&D Systems, Inc. Minneapolis, MN, USA). Standard curves were generated for each ELISA plate used. The samples were analyzed in duplicate with a KHB ST-360 micro-plate Reader (Shanghai Kehua Bio-Engineering Co.,Ltd.)

### Statistical analysis

Statistical analyses were done with SPSS software version 17 and MedCalc version 12.7.0.Differences between the four study groups were assessed by the Kruskal-Wallis nonparametric Test, and differences between two groups by the Mann-Whitney nonparametric U Test. ROC curves were plotted for each biomarker to investigate their capability to distinguish between HCC and non-HCC, and moreover define the cut-off value of each biomarker for HCC diagnosis by maximum sensitivity and specificity [14]. Prediction of HCC and AFP-negative HCC from LC patients by independent variables was assessed in univariate and multivariate analyses with binary logistic regression. Correlations between the investigated parameters were investigated using Pearson’s correlation coefficient. The difference in rates was evaluated by chi-square test.

To assess whether the combined use of DKK1, TEMs and AFP measurements was better than either of these three biomarkers alone, binary logistic regression analysis was further performed to create a new variable predicted probability (*P1*) for HCC on the basis of the following equation (HCC group versus LC, CHB and NC groups): Logit *P1* = -2.635+0.002×DKK1+0.001×AFP+0.147×TEMs.

The reported *P*-values are results of two-sided tests. *P*-values <0.05 are considered statistically significant.

## Results

### TEMs and DKK1 are significantly increased in HCC patients

To investigate the diagnostic performance of DKK1 and TEMs for the diagnosis of HCC, plasma DKK1 level and the percentage of TEMs in peripheral CD14+CD16+ monocytes were analyzed by ELISA and flow cytometry, respectively. As shown in Table 2, the median percentage of TEMs in peripheral CD14+CD16+ monocytes and the median plasma DKK1 level in HCC group were significantly higher than those in LC, CHB and NC groups (all p <0.05). Comparisons of DKK1 and TEMs between the45 HCC patients with AFP-negative and 37 HCC patients with AFP-positive were analyzed. We did not detect any significant difference. Similar results were also found between the HCC patients with DKK1 and TEMs low and high groups as defined by the cut-off (as shown in S1 Table).

**Table 2:** Plasma DKK1 level and the percentage of TEMs in different groups.

Groups	Patients(n)	DKK1 median(IQR), ng/L	*P* value	TEMs median(IQR),%	*P* value
Total HCC	82	780.70 (619.68–1171.96)	0.022*	8.25 (3.77–13.45)	0.012*
AFP negative HCC	45	771.44 (642.45–933.07)	0.083^#^	8.11 (5.59–12.40)	0.006*
LC	29	686.91 (467.80–863.58)	0.188^##^	4.77 (2.36–9.43)	0.350^##^
CHB	28	570.88 (248.83–904.45)	0.123^###^	3.48 (2.21–5.91)	0.564^###^
NC	31	432.83 (218.84–658.49)	0.003*	3.54 (1.85–5.73)	0.126^#^

*P<0.05 (vs LC)

^#^*P*≥0.05 (vs LC)

^##^ *P*≥0.05 (vs CHB)

^###^ *P*≥0.05 (vs NC).

HCC, hepatocellular carcinoma; LC, liver cirrhosis; CHB, chronic hepatitis B virus infection; NC, normal controls; IQR, Inter-Quartile Range. Kruskal-Wallis H test was used to analyzed the statistical significance of DKK1 and TEMs between total patients, all *P*<0.0001. All *P* values were analyzed by Mann-Whitney nonparametric U test.

In addition, the median percentage of TEMs in peripheral CD14+CD16+ monocytes and the median plasma DKK1 level in the 45 AFP-negative HCC patients were analyzed. It was found that the median percentage of TEMs in peripheral CD14+CD16+ monocytes in these patients were significantly higher than that in LC, CHB and NC groups (all *P*<0.05). Althoughplasma DKK1 level could be used to distinguish AFP-negative HCC patients from chronic HBV infected patients and healthy controls (all *P*<0.05), we did not detect any significant difference between AFP-negative HCC and LC patients (*P* = 0.083).

### Comparison of AUC, sensitivity and specificity of TEMs, DKK1 and AFP in distinguishing HCC patients from the other control groups

**Fig 2:**
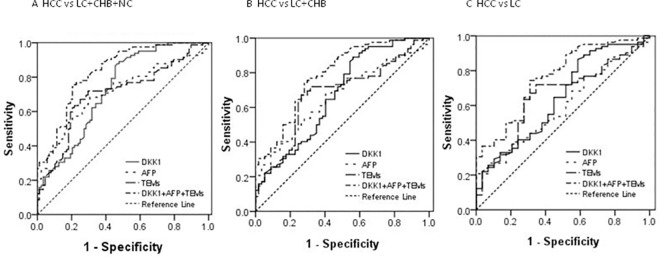
ROC curves for DKK1, TEMs, AFP and Logit *P*1 for the diagnosis of HCC versus different cohorts. **(**A) ROC curves for DKK1, TEMs, AFP and Logit *P*1 for the diagnosis of HCC versus all controls, with an AUC value of 0.728, 0.701, 0.712 and 0.833 respectively. (B) ROC curves for DKK1, TEMs, AFP and Logit*P*1 for the diagnosis of HCC versus LC and CHB controls, with an AUC value of 0.673, 0.673, 0.664 and 0.789 respectively. (C) ROC curves for DKK1, TEMs, AFP and Logit*P*1 for the diagnosis of HCC versus LC controls, with an AUC value of 0.644, 0.657, 0.592 and 0.762 respectively. ROC = receiver operating characteristic. AUC = area under receiver operating characteristic. HCC = hepatocellular carcinoma. CHB = chronic hepatitis B virus infection. LC = liver cirrhosis. Logit *P1* = -2.635+0.002×DKK1+0.001×AFP+0.147×TEMs.

We next analyzed the ROC curves to evaluate the sensitivity and specificity of DKK1, TEMs, AFP and Logit *P*1 in the diagnosis of HCC patients versus the other three cohorts (Fig 2). The ROC curves showed that the optimal diagnostic cutoff value was 550.93 ng/L for DKK1 and 4.95% for TEMs. We chose 20 μg/L as the cutoff value for AFP in this study.

As shown in Fig 2A, Tables 3 and 4, the AUC showed no significant difference between DKK1, TEMs and AFP. In combination, the AUC for the new variable Logit *P*1 of binary logistic regression analysis of triple markers (DKK1, TEMs and AFP) showed the biggest AUC (all *P*-values<0.05). As a single marker, DKK1>550.93 ng/L showed the highest sensitivity (89.02%) and the lowest specificity (52.27%), whereas AFP>20 μg/L showed the lowest sensitivity (45.12%) and the highest specificity (85.23%).

**Table 3:** Measurement results of DKK1, TEMs or AFP alone and their combinations for HCC diagnosis in different cohorts.

Markers	AUC(95% CI)	Sn(%)	Sp(%)	Sn+Sp	PPV(%)	NPV(%)
HCC vs LC,CHB and NC						
DKK1	0.728(0.654–0.793)	89.02	52.27	1.41	63.48	83.64
TEMs	0.701(0.626–0.768)	71.95	70.45	1.42	69.41	72.94
AFP	0.712(0.638–0.779)	45.12	85.23	1.30	74.00	62.50
DKK1+TEMs+AFP	0.833(0.768–0.886)	76.83	77.27	1.54	75.90	78.16
HCC vs LC and CHB						
DKK1	0.673(0.588–0.752)	89.02	43.86	1.33	69.52	73.53
TEMs	0.673(0.589–0.750)	71.95	68.40	1.40	76.62	62.90
AFP	0.664(0.579–0.742)	45.12	77.19	1.22	74.00	49.44
DKK1+TEMs+AFP	0.789(0.711–0.853)	76.83	68.42	1.45	77.78	67.24
HCC vs LC						
DKK1	0.664(0.548–0.733)	89.02	37.93	1.27	80.22	55.00
TEMs	0.657(0.561–0.745)	71.95	65.52	1.37	85.51	45.24
AFP	0.592(0.495–0.685)	45.12	65.52	1.11	78.72	29.69
DKK1+TEMs+AFP	0.762(0.672–0.838)	76.83	62.07	1.39	85.14	48.65
AFP-negative HCC vs LC,CHB and NC						
DKK1	0.709(0.624–0.795)	91.11	52.27	1.43	49.40	92.00
TEMs	0.739(0.648–0.829)	80.00	70.45	1.50	58.06	87.32
DKK1+TEMs	0.785(0.709–0.861)	40.00	82.95	1.23	54.55	73.00
AFP-negative HCC vs LC and CHB						
DKK1	0.649(0.542–0.755)	91.11	43.86	1.35	56.16	86.21
TEMs	0.708(0.605–0.812)	80.00	68.42	1.48	66.67	81.25
DKK1+TEMs	0.729(0.632–0.827)	40.00	77.19	1.57	58.06	61.97
AFP-negative HCC vs LC						
DKK1	0.620(0.483–0.757)	91.11	37.93	1.29	69.49	73.33
TEMs	0.692(0.564–0.819)	80.00	65.52	1.46	78.26	67.86
DKK1+TEMs	0.707(0.577–0.837)	40.00	72.41	1.12	69.23	43.75

**Table 4:** Assessment of AUC values of DKK1, TEMs or AFP alone and their combinations in distinguishing HCC or AFP-negative HCC and other cohorts.

	*P*	*P*	*P*
	HCC vs all controls	HCC vs LC+CHB	HCC vs LC
AFP vs DKK1	0.772	0.896	0.575
AFP vs TEMs	0.839	0.895	0.462
DKK1 vs TEMs	0.620	0.993	0.876
Logit *P*1 vs AFP	0.006	0.017	0.029
Logit *P*1 vs DKK1	0.000	0.001	0.018
Logit *P*1 vs TEMs	0.000	0.004	0.021
	AFP-negative HCC vs all controls	AFP-negative HCC vs LC+CHB	AFP-negative HCC vs LC
DKK1 vs TEMs	0.621	0.392	0.398
Logit *P*2 vs DKK1	0.012	0.032	0.101
Logit *P*2 vs TEMs	0.204	0.596	0.737

Logit *P1* = -2.635+0.002×DKK1+0.001×AFP+0.147×TEMs, Logit *P*2 = -2.565+0.002×DKK1+0.137×TEMs.

### TEMs rather than DKK1 could serve as a complementary biomarker for AFP in the differential diagnosis of AFP-negative HCC versus LC patients

**Fig 3:**
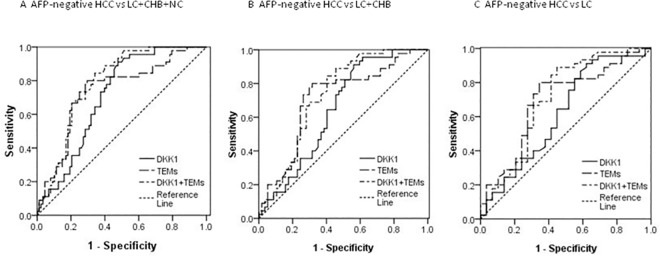
ROC curves for DKK1, TEMs and Logit *P*2 for the diagnosis of AFP-negative HCC versus different cohorts. **(**A) ROC curves for DKK1, TEMs and Logit*P*2 for the diagnosis of HCC versus all controls, with an AUC value of 0.709, 0.739 and 0.785 respectively. (B) ROC curves for DKK1, TEMs and Logit*P*2 for the diagnosis of HCC versus LC and CHB controls, with an AUC value of 0.649, 0.708 and 0.729 respectively. (C) ROC curves for DKK1, TEMs and Logit*P*2 for the diagnosis of HCC versus LC controls, with an AUC value of 0.620, 0.692 and 0.707 respectively. ROC = receiver operating characteristic. AUC = area under receiver operating characteristic. HCC = hepatocellular carcinoma. CHB = chronic hepatitis B virus infection. LC = liver cirrhosis. Logit *P*2 = -2.565+0.002×DKK1+0.137×TEMs.

In addition, in the 45 AFP-negative HCC patients, their AUC for DKK1 and TEMs for HCC diagnosis from the other three control groups was 0.709 (95% CI 0.624–0.795, Fig 3A, Table 3, *P<*0.01) and 0.739 (95% CI 0.648–0.829, Fig 3A, Table 3, *P<*0.01) respectively. However, the AUC for DKK1 was only 0.620 (95% CI 0.483–0.757, Fig 3C, Table 3, *P* = 0.083) in differentiating AFP-negative HCC from LC. When the cutoff value was selected at 550.93 ng/L, its sensitivity was 89.11%, but the specificity was only 37.93%. The AUC for TEMs was 0.692 (95% CI 0.564–0.819, Fig 3C, Table 3, *P*<0.01) in differentiating AFP-negative HCC from LC. Its sensitivity and specificity were 80.0% and 65.52% respectively.

To assess whether the combined use of DKK1 and TEMs measurements was better than either of these two biomarkers alone, binary logistic regression analysis was further performed to create a new variable predicted probability (*P2*) for AFP-negative HCC on the basis of an equation (AFP-negative HCC versus all LC, CHB and NC groups): Logit *P*2 = -2.565+0.002×DKK1+0.137×TEMs. Although the combined use of DKK1 and TEMs increased the AUC (0.785; 95% CI 0.709–0.861, Fig 3A) in differentiating AFP-negative HCC from LC, chronic HBV infected patients and normal controls, the combination use of DKK1 and TEMs was no better than TEMs alone in differentiating AFP-negative HCC from the other three control cohorts, and the combined use of DKK1 and TEMs did not statistically increased the AUC (0.707; 95% CI 0.577–0.837, Fig 3C) as compared with DKK1 or TEMs alone in differentiating AFP-negative HCC from LC (Fig 3C, Table 4).

Univariate and multivariate analyses by binary logistic regression were further performed to assess the significance of each marker in prediction of HCC and AFP-negative HCC from LC patients. Univariate analysis revealed DKK1 (*P* = 0.043, odds ratio = 0.999) and TEMs (*P* = 0.018, odds ratio = 0.901) as predictors for HCC differentiating from LC patients. In multivariate analysis, Only TEMs (*P* = 0.011, odds ratio = 0.892) prevailed as a valuable predictor for HCC differentiating from LC patients. Only TEMs prevailed as a significant predictor for AFP-negative HCC differentiating from LC patients in univariate and multivariate analyses (*P* = 0.016, *P* = 0.023). (as shown in S2 Table).

### There were no or weak correlation between plasma DKK1, AFP and the percentage of TEMs in peripheral CD14+CD16+ monocytes

**Fig 4:**
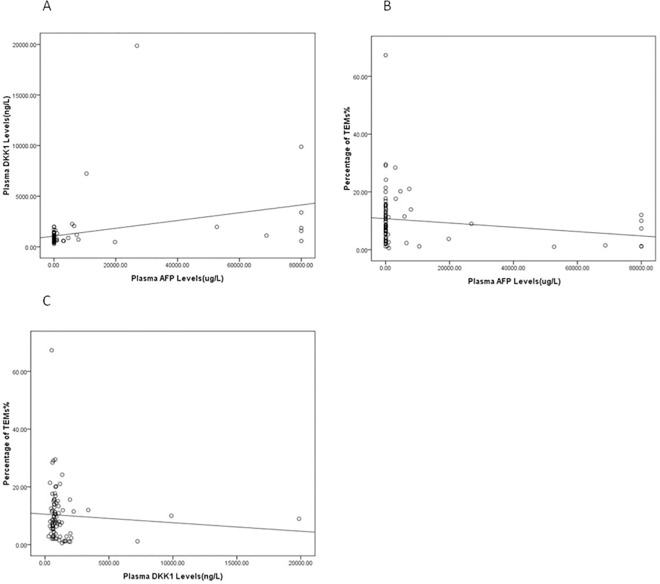
There were no or weak correlations between plasma DKK1, AFP and the percentage of TEMs. Correlations between the percentage of TEMs, DKK1 and AFP were analyzed in 82 HCC patients. (A) Pearson’s analysis shows a low positive correlation between plasma DKK1 and AFP(n = 82, *P* = 0.002, r = 0.332). (B) Pearson’s analysis shows no correlation between the percentage of TEMs in peripheral CD14+CD16+ monocytes and AFP (n = 82, *P* = 0.129, r = -0.169). (C) Pearson’s analysis shows no correlation between the percentage of TEMs in peripheral CD14+CD16+ monocytes and DKK1 (n = 82, *P* = 0.489, r = -0.078).

The correlations between plasma DKK1, AFP and the percentage of TEMs in peripheral CD14+CD16+ monocytes were analyzed in 82 HCC patients. It was found that there was a weak correlation between plasma DKK1 and AFP level (*P* = 0.002, r = 0.332; Fig 4A), Neither the plasma AFP level (*P* = 0.129, r = -0.169; Fig 4B) nor DKK1 (*P* = 0.489, r = -0.078; Fig 4C) was correlated with the percentage of TEMs in peripheral CD14+CD16+monocytes. These results were consistent with the results of S1 Table.

## Discussion

TEMs are a subgroup of circulating and tumor-infiltrating myeloid cells with potency to promote angiogenesis in xenotransplanted human tumors [6]. Previous studies [9, 15] defined TEMs as CD14+CD16+Tie2+ cells and reported that they were increased in HCC patients.TEMs are generally considered as a novel biomarker and potential therapeutic target for the diagnosis of HCC. However, reports about TEMs in healthy individuals have been controversial [6, 15]. In addition, the procedure of TEM analysis is complex, usually requiring as much as 10ml peripheral whole blood to isolate individual mononuclear cells by using Ficoll-Hypaque density gradient centrifugation[15]. In this study, we evaluated TEMs by using direct immunfluorescence staining as described in previous studies [6,13]. As expected, the percentage of TEMs in peripheral blood CD14+CD16+ monocytes of HCC patients was significantly increased as compared with that in healthy controls and patients with HBV-related CHB and LC. A previous study[15] reported that the median percentage of TEMs in peripheral blood CD14+CD16+ monocytes of NC group was 23.4%, which was significantly higher than that obtained in the present study (4.33%, IQR 1.85–5.73%). In addition, He et al showed that the AUC (0.800, 95% CI 0.704–0.896) for TEMs for HCC diagnosis from HBV-related LC was superior to our result (0.657, 95% CI 0.561–0.745). We hypothesize that the possible reason may be due to the different manufacturers of the Tie2+ antibody used and the different operating methods. In addition, different statistic populations and potential statistic biases may also lead to such differences.

As shown in Table 3, DKK1 was the most sensitive marker for HCC in different cohorts.Previous studies demonstrated that DKK1 was a biomarker for early diagnosis of HCC. Shen et al [10] reported that the median serum DKK1 concentration was 3.08 (IQR 1.75–4.57) ng/mL in HCC patients, with an optimal diagnostic cutoff of DKK1 of 2.153 ng/mL. This median serum DKK1 value was higher than that in the present study. The reason may be that we used plasma rather than serum for the measurement due to sample availability. According to the operating protocol of the kit used in this study, the serum level of DKK1 was higher than the plasma level of DKK1 due to platelet releasing[16,17]. Janget al [18] reported that the cutoff and median plasma DKK1 were 500 and 1497.1 (IQR 279.0–782.1) pg/mL in HCC patients, respectively, which are consistent with 550.93 and 1351.89 (IQR 619.68–1171.96) ng/L in our study. Shen et al [10] reported that the AUC, sensitivity and specificity of DKK1 in differentiating between LC, chronic HBV infected patients and healthy controls were 0.848, 69.1% and 90.6% respectively versus 0.830, 57.8% and 88.0% for AFP. However, their results were not reproducible in this study. In this study although DKK1 had the highest sensitivity (89.02%), the specificity (52.27%) is lower than AFP (85.23%). Combination of DKK1, AFP and TEMs could significantly increase the AUC (0.833; 95% CI 0.768–0.886) for HCC diagnosis, which is higher than that when any of the biomarkers was used alone. However, the sensitivity was lower than that of DKK1(χ^2^ = 4.307,P = 0.038), and the specificity was lower than that of AFP(χ^2^ = 12.05,P = 0.001).

Even with the help of advanced imaging technology, detection of early-stage and AFP-negative HCC remains difficult. A previous study [10] reported that the AUC, sensitivity and specificity of DKK1 were 0.830 (0.785–0.875), 70.4% and 84.7% in differentiating AFP-negative HCC from LC, and chronic HBV infected patients, which are superior to the results reported in the present study. As shown in Table 2 of the present study, although plasma DKK1 level could distinguish AFP-negative HCC from chronic HBV infected patients and healthy controls, it was unable to distinguish AFP-negative HCC from LC patients. Similarly, Jang et al [18] showed that the AUC of DKK1 was 0.617 in differentiating AFP-negative HCC from LC controls, which is consistent with our result (0.620, 95% CI 0.483–0.757).

We also firstly evaluated whether TEMs could be used as a marker for the diagnosis of AFP-negative HCC. The AUC for TEMs was 0.739 in differentiating AFP-negative HCC from LC, chronic HBV infected patients and healthy controls (95% CI 0.648–0.829). The AUC for TEMs was 0.692 in differentiating AFP-negative HCC from LC patients (95% CI 0.564–0.819), with a sensitivity of 80.0% and a specificity of 65.52%. Only TEMs prevailed as a significant predictor for AFP-negative HCC differentiating from LC patients in univariate and multivariate analyses (*P* = 0.016, *P* = 0.023).

We also found that there were no or weak correlations between plasma DKK1, AFP and the percentage of TEMs in peripheral CD14+CD16+ monocytes, suggesting that DKK1, TEMs and AFP could complement each other for screening and diagnosing HCC.

In conclusion, the combination of TEMs, DKK1 and AFP showed a larger AUC than each biomarker alone. TEMs and DKK1 could be complementary to AFP in the diagnosis of HCC. Only TEMs but not DKK1 could be a complementary marker to AFP in diagnosing AFP-negative HCC versus LC patients.

## Supporting information

S1 Table Comparisons of other markers among the three indicators negative and positive groups. (DOCX)

S2 Table Univariate and multivariate analyses by binary logistic regression to assess the prediction of HCC and AFP-negative HCC from LC patients. (DOCX)
