# Supplementary material for: TEMs but not DKK1 could serve as complementary biomarkers for AFP in diagnosing AFP-negative hepatocellular carcinoma
Source: PLoS One. 2017 Sep 13;12(9):e0183880. doi: 10.1371/journal.pone.0183880 (PMC5597119; doi:10.1371/journal.pone.0183880)
Supplement: S1 Table — (DOCX) [file pone.0183880.s001.docx]

**S1Table.Comparisons of other markers among the three indicators negative and positive groups.**

| Markers | Patients(n) | AFP median(IQR), ug/L | *P* value | DKK1 median(IQR), ng/L | *P* value | TEMs median(IQR),% | *P* value |
| --- | --- | --- | --- | --- | --- | --- | --- |
| TEMs |  |  |  |  |  |  |  |
| ＞4.95 | 59 | 8.44 (3.93- 94.00) | 0.074 | 758.80(614.50- 968.98) | 0.05 | - | - |
| ≤4.95 | 23 | 121.95(4.07- 10520.76) |  | 1108.54(665.67- 1686.59) |  | - | - |
| DKK1 |  |  |  |  |  |  |  |
| ＞550.93 | 73 | 12.87 (3.82- 916.47) | 0.982 | - | - | 8.39 (3.50- 13.60) | 0.619 |
| ≤550.93 | 9 | 21.02 (4.15- 43.64) |  | - | - | 8.02(4.60- 16.98) |  |
| AFP |  |  |  |  |  |  |  |
| ＞20.00 | 37 | - | - | 805.09(602.21- 1735.42) | 0.278 | 8.75(2.71- 14.80) | 0.867 |
| ≤20.00 | 45 | - | - | 771.44(642.45- 933.07) |  | 8.11(5.59- 12.40) |  |

*P* values were analyzed by Mann-Whitney nonparametric U test.
