# Supplementary material for: TEMs but not DKK1 could serve as complementary biomarkers for AFP in diagnosing AFP-negative hepatocellular carcinoma
Source: PLoS One. 2017 Sep 13;12(9):e0183880. doi: 10.1371/journal.pone.0183880 (PMC5597119; doi:10.1371/journal.pone.0183880)
Supplement: S2 Table — (DOCX) [file pone.0183880.s002.docx]

**S2 Table. Univariate and multivariate analyses by binary logistic regression to assess the prediction of HCC and AFP-negative HCC from LC patients.**

| Markers | Univariate Analysis | | | Multivariate Analysis | | |
| --- | --- | --- | --- | --- | --- | --- |
|  | *P* | Odds Ratio | 95% CI | *P* | Odds Ratio | 95% CI |
| HCC vs LC | | | | | | |
| DKK1 | 0.043 | 0.999 | 0.997- 1.000 | 0.102 | 0.999 | 0.997- 1.000 |
| TEMs | 0.018 | 0.901 | 0.826- 0.982 | 0.011 | 0.882 | 0.801- 0.972 |
| AFP | 0.210 | 1.000 | 0.999- 1.000 | 0.448 | 1.000 | 0.999- 1.000 |
| AFP-negative HCC vs LC | | | | | | |
| DKK1 | 0.119 | 0.999 | 0.997- 1.000 | 0.181 | 0.999 | 0.997- 1.001 |
| TEMs | 0.016 | 0.876 | 0.786- 0.976 | 0.023 | 0.882 | 0.792- 0.983 |
